# Consensus Statement: Toward Opioid-Free Arthroplasty: A Leadership Forum

**DOI:** 10.1007/s11420-018-09664-w

**Published:** 2019-02-01

**Authors:** Seth Waldman, Charles N. Cornell, Louis A. Shapiro, Todd J. Albert, William Schairer, E. Carlos Rodriguez-Merchan, Ellen M. Soffin, Christopher Lee Wu, Mark Barnes, Alex Rich, Jonathan Avery, Travis N. Rieder

**Affiliations:** 1 Pain Management Division, Department of Anesthesiology, Hospital for Special Surgery, 535 East 70th Street, New York, NY 10021 USA; 2 Department of Orthopaedic Surgery, Hospital for Special Surgery, New York, NY USA; 3 Department of Orthopedic Surgery, Weill Cornell Medicine, New York, NY USA; 4 Executive Leadership, Hospital for Special Surgery, New York, NY USA; 5 Department of Orthopaedic Surgery, La Paz University Hospital, Madrid, Spain; 6 Department of Anesthesiology, Hospital for Special Surgery, New York, NY USA; 7 Department of Anesthesiology, Weill Cornell Medicine, New York, NY USA; 8 Armstrong Institute for Patient Safety and Quality, Johns Hopkins University, Baltimore, MD USA; 9 Ropes and Gray LLP, Boston, MA USA; 10 Carolina Health Informatics Program, University of North Carolina, Chapel Hill, NC USA; 11 Department of Psychiatry, Weill Cornell Medicine, New York, NY USA; 12 Berman Institute of Bioethics, Johns Hopkins University, Baltimore, MD USA

## Introduction

**Fig. 1:**
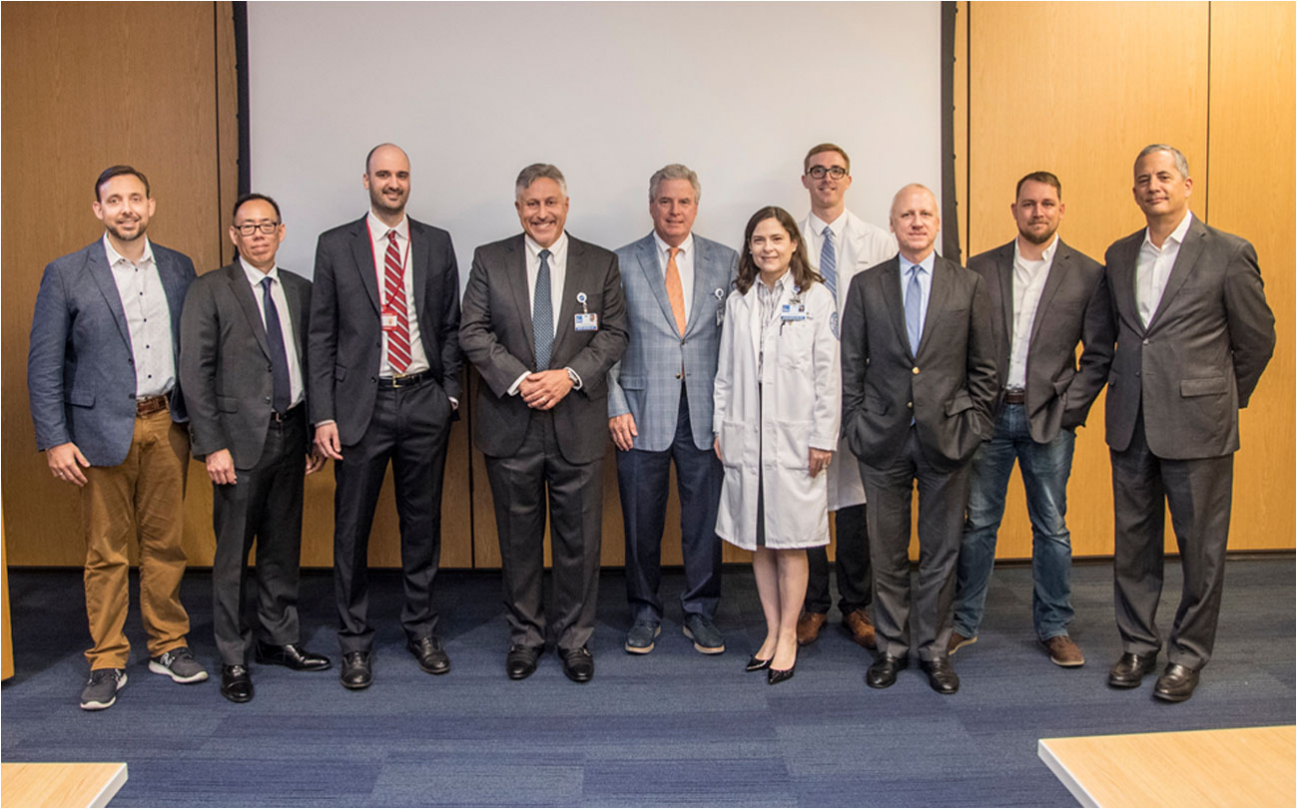
Participants in *Toward Opioid-Free Arthroplasty: A Leadership Forum*, June 1, 2018, at Hospital for Special Surgery (pictured left to right): Travis N. Rieder, PhD, Christopher Lee Wu, MD, Jonathan Avery, MD, Seth Waldman, MD, Charles N. Cornell, MD, Ellen M. Soffin, MD, PhD, William Schairer, MD, Mark Barnes, JD, LLM, Alex Rich, MPH, and Louis A. Shapiro, FACHE. (Not pictured: Todd J. Albert, MD, and E. Carlos Rodriguez-Merchan, MD, PhD.) Photograph by Robert Essel.

As part of ongoing efforts to address the opioid crisis, on June 1, 2018, HSS Journal^®^ convened a multidisciplinary panel of experts for a daylong discussion: *Toward Opioid-Free Arthroplasty: A Leadership Forum*. Each invited participant presented on a specific aspect of the opioid crisis and/or pain management as it relates to total hip or knee arthroplasty. Representing orthopedics, anesthesiology, psychiatry, bioethics, health information technology, and law, this group crafted a consensus statement on appropriate pain management for total hip or knee arthroplasty. Many have also contributed to this special issue of HSS Journal on “Opioid Prescribing and Pain Management” (February 2019) (Fig. 1).

This consensus statement focuses on pre-operative, peri-operative, and post-operative strategies at institutional and individual levels. It is not designed necessarily to guide clinicians in eliminating opioid prescribing but rather to aid care teams in providing the best and safest post-surgical care possible.

The authors acknowledge that pain management amid the opioid epidemic is a shifting landscape and have arrived at this consensus as of December 2018. They offer it with the understanding that determining the best care for each patient requires ethical decision-making—weighing the risks and benefits of various pain-management strategies, including opioids.

## Prescribers and Clinical Teams Must Identify At-risk Patients

- Pre-operatively, determine a patient’s need for a complex pain evaluation and opioid-management plan by obtaining a detailed history of chronic and acute pain, opioid use, and other substance use.
- Confirm the clinician who has responsibility for each patient’s pain-management plan, including determining medical need, ordering urine toxicity screening when appropriate, and checking state prescription drug monitoring databases.

## Prescribers and Institutions Must Address Risk Stratification and Mitigation

- Understand that the risks of opioid use after total joint replacement include not only dependency, addiction, and hyperalgesia (i.e., worsened pain) but also an increased possibility of complications (e.g., revision surgery).
- Improve risk assessment by educating clinical staff on issues related to substance use disorder.
- Implement risk-mitigation strategies, such as providing naloxone rescue kits for patients requiring high opioid doses and limiting initial dosage of discharge prescriptions.

## Clinicians Must Establish Opioid-Responsible Prescribing and Education

- Institute an opioid-responsible pain-management strategy for each patient. This involves identifying a single prescriber who uses evidence-based recommendations for prescribing opioids conservatively—i.e., for the shortest duration and at the lowest dose to manage that individual’s peri-operative pain—and a plan for weaning while avoiding withdrawal symptoms.
- Provide easy-to-understand fact sheets for patients that review the risks and benefits of opioid use, weaning, and safe storage and disposal (provide pill deactivation kits or locations for safe disposal, as well).
- Commit to patient and family to reduce the risks of opioid use and manage withdrawal symptoms should they arise. Provide contact information of the prescriber whom patient, family members, or team members may call with questions.
- Establish institutional standards to guide the use of opioids, ensuring quality improvement and monitoring in clinical practice and in the education and training of physicians, pharmacists, nurses, and other professional staff.
- Refer patients when indicated for appropriate treatment of substance use disorder.

## Institutions Should Align Communications Among Patients and Practitioners

- Script uniform messages for clinicians to use when discussing patients’ expectations for surgery, pain management, rehabilitation, and opioid use, and when responding to patients’ concerns about pain, dependency, hyperalgesia, and addiction.
- Maintain open communication with patients post-surgically to oversee pain management and opioid weaning. A patient who requests refills or reports increased or persistent pain deserves in-depth re-evaluation for additional pain relief or behavioral health assessment.
- Establish clear protocols and lines of communication for patient handoff among clinicians and settings.
- Review the execution of each patient’s pain-management plan as part of routine quality improvement. Team members can discuss successes and failures, and data analysts can evaluate metrics on successful weaning, referrals, and other data.
- Make use of an opioid agreement, which delineates the expectations and responsibility of both provider and patient, ensuring that the pain-management plan is thoroughly explained and understood.

## Institutions Should Ensure Non-opioid-based Comprehensive Pain Management

- Multimodal and regional anesthesia strategies are strongly recommended for total joint arthroplasty. These approaches have been shown to decrease opioid use, increase patient satisfaction, and shorten lengths of stay.
- Incorporate innovative technologies to measure both patient-reported outcomes and clinicians’ prescribing patterns. Such data are to be used for continuous quality improvement.

## Next Steps: Advance Understanding of Novel Analgesics, Techniques, and Care of the Opioid-Tolerant Patient

- Identify gaps in the evidence on post-arthroplasty pain management and alternatives to opioids.
- Develop comparative research studies on the methods for enhancing the quality and extending the duration of opioid-free post-arthroplasty analgesia.
- Determine the risk factors of and methods for preventing long-term opioid use in opioid-naïve patients and/or escalation of opioid use in opioid-tolerant patients undergoing hip or knee arthroplasty.
- Investigate the use of “comfort menus”—alternatives to opioids for pain relief such as massage therapy, aromatherapy, music, or visualization—in post-arthroplasty pain management.
- Establish opioid-use and pain-management protocols for all orthopedic surgical procedures.

## A Checklist for Prescribers of Opioid Analgesia After Total Knee or Hip Arthroplasty

- Is there a legitimate medical need for a prescribed opioid? If yes, state the need.
- Have appropriate non-opioid measures been implemented? If yes, list them.
- Has the patient’s risk been assessed?
  - The state prescription drug monitoring program (PDMP) database has been checked.
  - The patient’s history of substance use, mental health conditions, and sleep apnea has been documented.
  - Toxicology reports, if indicated, have been reviewed.
- Has the prescriber referred to the service-specific guideline when choosing a medication and dosage, adhering to the adage “start low, go slow”?
- Has the patient been educated on the risks and benefits of opioid use, including safe storage, disposal, and tapering?
- Has the patient received the name and phone number of the prescribing clinician?
- Have the patient and prescriber signed an opioid agreement?
- Has all of the above been documented?

## Electronic supplementary material

ESM 1 (PDF 1.19 mb)

ESM 2 (PDF 1.19 mb)

ESM 3 (PDF 1.19 mb)

ESM 4 (PDF 1.19 mb)

ESM 5 (PDF 1.19 mb)

ESM 6 (PDF 1.19 mb)

ESM 7 (PDF 1.19 mb)

ESM 8 (PDF 1.19 mb)

ESM 9 (PDF 1.21 mb)

ESM 10 (PDF 1.19 mb)

ESM 11 (PDF 1.19 mb)

ESM 12 (PDF 1.19 mb)

